# Test-Retest Reliability of the Coronary Heart Disease Damp Phlegm and Blood Stasis Pattern Questionnaire: Results from a Multicenter Clinical Trial

**DOI:** 10.1155/2021/6291301

**Published:** 2021-11-16

**Authors:** Ge Fang, Yaxin Wang, Zhenqian Yan, Xiaowen Zhou, Xingyu Fan, Xiaoqian Liao, Zhixi Hu, Xiantao Li

**Affiliations:** ^1^College of Traditional Chinese Medicine, Hunan University of Chinese Medicine, Changsha 410208, China; ^2^School of Basic Medical Science, Guangzhou University of Chinese Medicine, Guangzhou 510006, China; ^3^Institute of Chinese Medicine Diagnosis, Hunan University of Chinese Medicine, Changsha 410208, China

## Abstract

**Background:**

Damp phlegm and blood stasis pattern (DPBSP) is the main pattern in coronary heart disease (CHD) patients. To quantify and standardize the diagnosis of DPBSP, questionnaires are usually administered. The CHD Damp Phlegm and Blood Stasis Pattern Questionnaire (CHD-DPBSPQ) is the standard metric for measuring CHD-DPBSP signs and symptoms in practice and clinical research. The CHD-DPBSPQ has moderate diagnostic efficiency, as evidenced by its receiver operating characteristic curves. Furthermore, and high reliability and validity have been shown in some studies but not in a multicenter clinical trial. Our purpose was to evaluate the test-retest reliability of a proprietary CHD-DPBSPQ.

**Methods:**

The CHD-DPBSPQ uses a standard procedure for measuring symptoms. The (interrater) reliability and validity of this questionnaire have been previously studied. Here, we evaluated the test interval and weighted kappa value of items of test-retest (intrarater) reliability of the CHD-DPBSPQ. The test-retest reliability was evaluated by the intraclass correlation coefficient (ICC) for the total CHD-DPBSPQ score and the phlegm domain and blood stasis domain scores. Weighted kappa statistics were calculated for the individual CHD-DPBSPQ items.

**Results:**

Using the CHD-DPBSPQ, 79 patients with late-stage CHD who were participating in a multicenter clinical trial were assessed twice. The ICCs for the CHD-DPBSPQ score were as follows: 0.827 for the total CHD-DPBSPQ, 0.778 for the phlegm domain score, and 0.828 for the blood stasis domain score. The reliability was slightly better in patients whose test interval was ≤14 days. The weighted kappa values of individual items showed moderate consistency.

**Conclusions:**

The CHD-DPBSPQ was found to have excellent test-retest reliability in this sample of patients.

## 1. Introduction

Coronary heart disease (CHD) has been the main cause of mortality and disability in both developed and developing countries in the last 20 years [1]. According to the Report on Cardiovascular Health and Diseases in China 2019, there were approximately 11 million CHD patients who were more than 15 years old in China [2]. In Western countries, CHD accounted for about around a third of all the deaths in people aged above 35, although CHD mortality has gradually declined [3]. This may be related to increased serum cholesterol levels caused by sex, age, hyperlipidemia, hypertension, diabetes, obesity, smoking, and other changes [4–8]. The prevalence of CHD is expected to continue to increase with contemporary lifestyles. Therefore, we need to rapidly strengthen the diagnostic criteria of CHD and provide better treatment for clinical patients.

Epidemiological investigations of the CHD syndrome have shown that the distributional characteristics are mainly blood stasis and phlegm turbidity. For example, Mao et al. found that the main syndrome characteristics of CHD from 1970 to 2010 were blood stasis, phlegm turbidity, and qi stagnation [9]. Wang et al. found that blood stasis, phlegm turbidity, and qi deficiency were the main syndrome elements of CHD by analyzing 115 cases of CHD diagnosed and treated by famous doctors [10].

The Coronary Heart Disease Damp Phlegm and Blood Stasis Pattern Questionnaire (CHD-DPBSPQ) is the standard metric for measuring CHD-DPBSP signs and symptoms in practice and clinical research [11–13]. The CHD-DPBSPQ was published in 2019 by combining an assessment of the damp phlegm pattern and blood stasis pattern in CHD Patients [14]. In other words, the CHD-DPBSPQ consists of two main domains that assess turbid phlegm and blood stasis [15]. According to the epidemiological investigation of Traditional Chinese Medicine (TCM) Syndromes of CHD in China from 1990 to 2020, the prevalence of blood stasis syndrome and phlegm turbidity syndrome amounted to 64.2% and 37.8%, respectively, and the two ranked in the top three syndromes [16].

TCM syndromes can be constructed into a set of standards and issued in the form of scales. Several studies have investigated the structure and metric properties of the CHD-DPBSPQ. The CHD-DPBSPQ has been shown to have a moderate diagnostic efficiency based on its receiver operating characteristic curves [12]. Some studies have shown that the CHD-DPBSPQ has high reliability and validity but the metric has yet to be assessed in a multicenter clinical trial [14]. The study's aim was to present research aimed at the test-retest reliability of the CHD-DPBSPQ in a multicenter clinical trial comprising CHD patients.

## 2. Materials and Methods

### 2.1. Subjects

The researchers had to identify patients who could participate in the questionnaire again to ensure that the number of respondents in the two surveys was five times the number of items in the questionnaire. In accordance with the study protocol, the inclusion criteria were as follows: the subjects were aged 18 years or above, provided informed consent for participation, had been diagnosed with CHD according to the guidelines [17, 18], and had been diagnosed with DPBSP by two experienced experts [19]. The exclusion criteria were as follows: patients with unstable angina and a diagnosis of diseases or syndromes other than CHD.

### 2.2. Raters

The raters were the main researchers. The raters were all experts in TCM or integrated Chinese and Western medicine in the treatment of cardiovascular diseases, with at least 20 years of clinical experience. All the raters explained the questionnaire to each patient before any evaluations were performed.

### 2.3. Cross-Sectional Validation of the Questionnaire

The questionnaire was validated in a cross-sectional, multicenter, observational, descriptive study that followed a test-retest design. The study was approved by the Research Ethics Committee of Tianjin University of Traditional Chinese Medicine (no. TJUTCMEC2015000). The construction definition, item generation, selection reliability, and validity of the CHD-DPBSPQ were performed according to standard procedures [20–22]. Next, we assessed the two domains in the questionnaire: the phlegm domain (chest distress, sleepiness, physical heaviness, obesity, sticky mouth, abdominal fullness, anorexia, greasy tongue fur, and slippery pulse) and the blood stasis domain (chest pain, cyanotic lips, dim complexion, dark purple tongue, petechiae or ecchymosis on the tongue, and sublingual vein cyanosis). The items were rated according to four response options: 0 = none, 1 = mild, 2 = moderate, and 3 = severe. Higher scores indicate a greater severity of the symptom in question.

### 2.4. Statistical Analysis

For the statistical analysis, SPSS version 25.0 (IBM SPSS Statistics for Windows, IBM Corp., Armonk, NY) and SAS software 9.4 (SAS Institute, Cary, NC) were used. The test-retest reliabilities of the different domains of the CHD-DPBSPQ were estimated by the intraclass correlation coefficient (ICC) [23]; the 95% confidence interval was also calculated. The test-retest reliability was estimated separately for the subdomains of subjects as defined by the number of days (with the 14th day as the dividing line). The test-retest reliability for single items was assessed by the weighted kappa statistics [24].

## 3. Results

**Figure 1:**
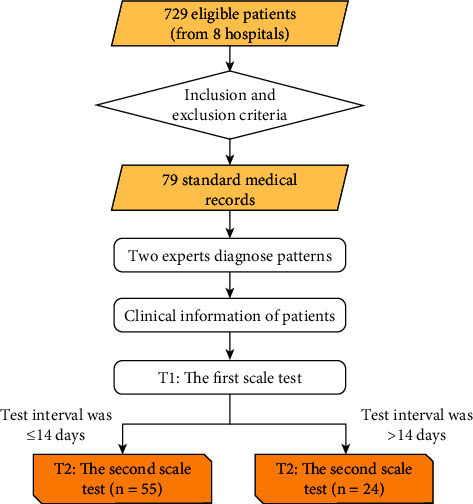
A flow diagram for the patients.

In our preliminary research, the data of 729 inpatients (from eight hospitals in the period between 2016 and 2018) were collected and screened from a doctor according to the aforementioned inclusion and exclusion criteria. Ultimately, two experienced CHD experts in TCM or integrated Chinese and Western medicine diagnostic patterns collected 79 inpatient data, which comprised the research sample. The specific data processing flow diagram that was designed is shown in Figure 1. The clinical characteristics of 79 CHD subjects with DPBSP (mean age: 65 years; most were males) were collected from different hospitals in China for the test-retest study (Table 1). All the patients had comorbidities, were of the Han ethnicity, and were married. The course of the disease was generally within 30 days. Patients with different severities of illness had different questionnaire scores.

**Figure 2:**
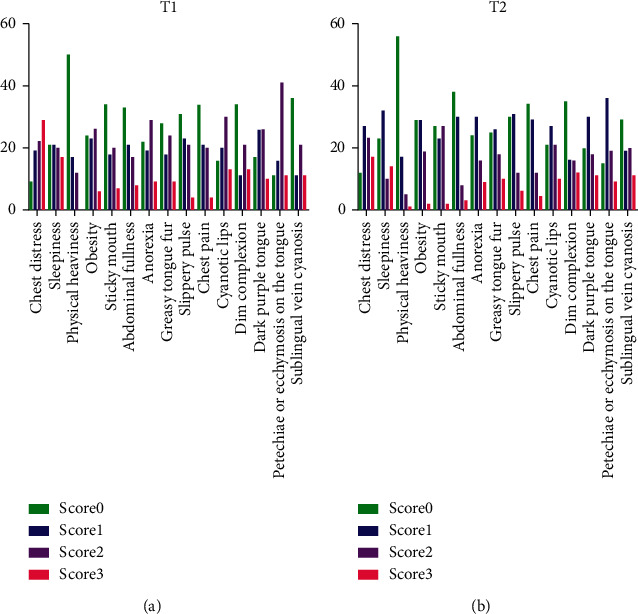
Frequency of the responses in the two-round scale test for each item. T1: the first scale test; T2: the second scale test.

Figure 2 shows the frequency of the four response options of the items in the first and the second scale tests. The ICCs for the test-retest reliability and related 95% confidence intervals are shown in Table 2. The ICCs were 0.827 for the total CHD-DPBSPQ, 0.778 for the phlegm domain, and 0.828 for the blood stasis domain. The ICCs for the patients whose test interval was >14 days were lower than those of the patients whose test interval was ≤14 days. The weighted kappa value of items was ≥0.4 for most items except abdominal fullness and greasy tongue fur, which were 0.3372 and 0.2238, respectively (Table 3).

## 4. Discussion

The CHD-DPBSPQ was developed and evaluated according to a set of standard procedures and then issued by the China Association of Chinese Medicine (CACM). To date, there has been no report of a cross-sectional study of TCM syndromes combined with a joint investigation of patients from multiple hospitals.

Our results showed that the total CHD-DPBSPQ and blood stasis domain had excellent test-retest reliabilities; the symptom-based subscales also showed considerably good reliability. However, the test-retest reliability of the phlegm domain was somewhat lower than that of the blood stasis domain of the CHD-DPBSPQ. This result may be because of the different characteristics of people in different regions, which reflect the so-called “treating the disease according to the individual condition” and “treating the disease according to the environment” aspects of TCM. However, the test-retest reliability of the phlegm domain was close to 0.75, which is the lower-limit indicator of good diagnostic tests. The ICCs were slightly better for patients with test-retest intervals ≤14 days than for those of patients with intervals >14 days. This finding is consistent with Andrew's report on the time interval [25]. The lower test-retest reliability of every CHD-DPBSPQ item can be explained by the fact that our sample of patients with >14 days of intervals generally achieved low scores on some items. Sleepiness (0.5602), physical heaviness (0.5194), sticky mouth (0.4662), anorexia (0.4256), chest pain (0.4332), dark purple tongue (0.4121), petechiae, or ecchymosis of the tongue (0.5298) items had weighted kappa values that were <0.60. Fleiss [26] suggested that the ICCs of items or questionnaires of <0.4 indicate a “poor” reliability. This result may be due to the particularity and clinical symptoms of TCM syndromes. The determination of a TCM syndrome is based on a series of syndrome groups. In clinical practice, even if patients show the damp phlegm and blood stasis pattern, the symptoms of these patients are not completely consistent. For example, “greasy fur” is common in China for people who are from the south but rare for those from the north.

Our study aimed to validate the test-retest reliability of the CHD-DPBSPQ in patients at multiple treatment centers in China. However, the results showed individual differences in the degree of the importance of the different items in the CHD-DPBSP. Even if the individual items of domains show low scores, these results are not necessarily reflected in the total CHD-DPBSPQ score. The CHD-DPBS syndrome is divided into two stages: phlegm in the early stage and blood stasis in the late stage. The average age of our patients was approximately 66 years, at which it is common for late-stage CHD patients to have “blood stasis” as the main stage, and the “phlegm” syndrome score is low. Deng [27] stated that phlegm is the initial stage of blood stasis, which further develops into blood stasis, which confirms the conclusion of the questionnaire. In general, the reliability score of the entire questionnaire was 0.827, which was >0.75, indicating that slight internal changes would not affect the conclusions drawn from the questionnaire.

In particular, one limitation of our study was the lack of early CHD-DPBSP patients. Thus, future studies should investigate patients from aged 18–66 years. Owing to medication effects, a substantial short-term variability in the CHD-DPBSPQ scores should be expected. Because this investigation comprised the basic work of diagnostic research, the present study lacks the “prescription-syndrome correspondence” to evaluate the reactive evidence of the diagnostic criteria of DPBSP in CHD. That is, we only selected CHD patients with DPBSP for measurement and excluded those DPBSP.

Nevertheless, our investigation has some strengths which are as follows. First, we assessed 79 patients to evaluate the test-retest reliability, which met the requirements of a sample size of >20 participants [28]. Second, the patients were from multiple centers in China. Third, our raters were all CHD clinical experts who were considerably familiar with the CHD-DPBSPQ. Finally, few reports exist on the test-retest reliability of the CHD-DPBSPQ; thus, the present research adds to the literature in this regard.

## 5. Conclusions

The CHD-DPBSPQ was found to be reliable and can be recommended for evaluations of CHD. The present study also showed that the CHD-DPBSPQ is a valid instrument for measuring the phlegm domain and blood stasis domain scores. The establishment of DPBSP provides a standard template for determining the test-retest reliability of TCM syndrome types and establishes a foundation for achieving TCM syndrome standardization.

## Figures and Tables

**Table 1:** Characteristics of the patients with CHD-DPBSP^*∗*^ (n = 79).

Variable	
Age (year)	67 ± 12.1
Male (%)	47 (60.5%)
Comorbidities (%)	68 (87.2%)
Han ethnicity (%)	79 (100%)
Marriage (%)	79 (100%)
Years since CHD diagnosis (days)	
≤30	40 (50.6%)
≤60	12 (15.2%)
≤90	7 (8.9%)
>90	20 (25.3%)
CHD-DPBSPQ (possible range)	
Total (0–45)	18.2 ± 9.2
Phlegm (0–27)	10.2 ± 5.4
Blood stasis (0–18)	8.0 ± 4.6

^*∗*^CHD-DPBSP was diagnosed according to the International Statistical Classification of Diseases and Related Health Problems 10th Revision (ICD-10).

**Table 2:** ICCs for the total CHD-DPBSPQ and subscales for all patients, those reassessed ≤14 days, and those reassessed >14 days after screening^*∗*^.

	All patients	Time of test-retest reliability	
		14 or 14 fewer days	More than 14 days
Total CHD-DPBSPQ	0.827 (0.729, 0.889)	0.897 (0.824, 0.940)	0.802 (0.542, 0.914)
Phlegm domain	0.778 (0.653, 0.858)	0.850 (0.742, 0.912)	0.734 (0.385, 0.885)
Blood stasis domain	0.828 (0.732, 0.890)	0.896 (0.821, 0.939)	0.808 (0.557, 0.917)

^*∗*^95% confidence intervals for the ICCs are given in brackets.

**Table 3:** Weighted kappa values for all CHD-DPBSPQ items^*∗*^.

Item	Weighted kappa
*Phlegm domain*	
Chest distress	0.3856 (0.2478, 0.5233)
Sleepiness	0.5602 (0.4060, 0.7144)
Physical heaviness	0.5194 (0.3850, 0.6537)
Obesity	0.6609 (0.5330, 0.7889)
Sticky mouth	0.4662 (0.3255, 0.6069)
Abdominal fullness	0.3372 (0.1784, 0.4960)
Anorexia	0.4256 (0.2660, 0.5853)
Greasy tongue fur	0.2238 (0.0781, 0.3696)
Slippery pulse	0.3920 (0.2338, 0.5501)
*Blood stasis domain*	
Chest pain	0.4332 (0.2773, 0.5890)
Cyanotic lips	0.6230 (0.4992, 0.7468)
Dim complexion	0.6094 (0.4819, 0.7369)
Dark purple tongue	0.4121 (0.2655, 0.5587)
Petechiae or ecchymosis on the tongue	0.5298 (0.3875, 0.6720)
Sublingual vein cyanosis	0.6830 (0.5603, 0.8057)

^*∗*^95% confidence intervals for the weighted kappa statistics are given in brackets.

## Data Availability

The datasets used and/or analyzed during the current study are available from the corresponding author on reasonable request.

## Abbreviations

CHD-DPBSPQ: Coronary Heart Disease Damp Phlegm and Blood Stasis Pattern Questionnaire
ICC: Intraclass correlation coefficient
CHD: Coronary heart disease
DPBSP: Damp phlegm and blood stasis pattern
TCM: Traditional Chinese medicine
ICD-10: The International Statistical Classification of Diseases and Related Health Problems 10^th^ Revision
CACM: China Association of Chinese Medicine.

## Additional Points

**Trial registration:** ChiCTR-OOC-15006599, registered on June 16, 2015, http://www.chictr.org.cn/showproj.aspx?proj=11233.
